# Uncovering plant epigenetics: new insights into cytosine methylation in rye genomes

**DOI:** 10.1093/jxb/erad144

**Published:** 2023-06-27

**Authors:** Navneet Kaur, Swapna Nayakoti, Natasha Brock, Nigel G Halford

**Affiliations:** Rothamsted Research, Harpenden, Hertfordshire AL5 2JQ, UK; Rothamsted Research, Harpenden, Hertfordshire AL5 2JQ, UK; Rothamsted Research, Harpenden, Hertfordshire AL5 2JQ, UK; Rothamsted Research, Harpenden, Hertfordshire AL5 2JQ, UK

**Keywords:** 5-Carboxycytosine, demethylation, epigenetics, 5-formylcytosine, 5-hydroxymethylcytosine, hydroxymethylation, 5-hydroxymethyluracil

## Abstract

This article comments on:

**Kalinka A, Starczak M, Gackowski D, Stępień E, Achrem M.** 2023. Global DNA 5-hydroxymethylcytosine level and its chromosomal distribution in four rye species. Journal of Experimental Botany **74**, 3488–3502.

**In plants, epigenetic modifications play an important role in the regulation of gene expression under different environmental conditions. Methylated cytosine (5mC) is a modification found in many eukaryotes, including plants. However, the synthesis and function of 5mC oxidation derivatives, which play important roles in mammalian systems, are still under investigation in plants. Kalinka *et al.* (2023) examined four species of rye (*Secale* spp.) to investigate the presence of 5-hydroxymethylcytosine (5hmC) and other 5mC oxidative derivatives. They verified the presence of these derivatives in rye genomes and demonstrated an association with coding regions of DNA, implying a possible role in plant genome regulation.**

Epigenetics is the study of phenotypic changes that result from alterations to an organism’s DNA, other than changes to the DNA sequence. Epigenetic mechanisms may affect gene activity, generating metastable epialleles that are differentially expressed in ostensibly genetically identical organisms. Plants have developed particularly sophisticated epigenetic mechanisms to regulate genes in response to environmental changes, probably due to their sessile nature. These mechanisms involve DNA methylation, histone modifications, small RNAs, and long non-coding RNAs.

## 5mC is a hallmark of epigenetic modification

The mechanisms of cytosine methylation and its inheritance are well established in plants (Takeda and Paszkowski, 2006; Muyle *et al.*, 2022). 5mC is a hallmark of epigenetic modification and has previously been associated with heterochromatin (regions of tightly packed DNA). It has been described as a fifth base of the genetic code in both plants and mammals (Lister and Ecker, 2009). 5mC is observed only at CG sites in mammals; however, plants methylate cytosines at CG, CHG, or CHH motifs (where H represents A, T, or C). Other modified bases, such as 5-hydroxymethylcytosine (5hmC), 5-formylcytosine (5fC), 5-carboxylcytosine (5caC), 5-hydroxymethyluracil (5hmU), and *N*^6^-methyladenine (6mA), are also reported to play critical roles in various biological processes in mammalian systems (Fu and He, 2012; Liu *et al.*, 2016).

The methylation/demethylation of cytosine maintains heterochromatin structure and genomic imprinting (where epigenetic differences affect the expression of genes that are inherited from the male or female parent), regulates gene expression, and activates or represses transposable elements. In mammals, 5hmC, 5fC, 5caC, and 5hmU are intermediates of DNA demethylation (Zhang *et al.*, 2018). 5hmC in particular has been shown to play an important role in the regulation of many cellular and developmental processes, such as pluripotency and neuron development (Ruzov *et al.*, 2011; Shi *et al.*, 2017), and is considered to be a marker of euchromatin (lightly packed DNA that is often being actively transcribed) (Wen *et al.*, 2014). There is no consistency between 5hmC abundance and gene expression levels; however, changes in the sum of 5mC and 5hmC in transcriptional regions have been shown to regulate transcription (Scholsberg *et al.*, 2019). In plants, on the other hand, there has hitherto been limited evidence supporting either the presence of 5hmC and other 5mC oxidation products or their possible epigenetic roles (Terragni *et al.*, 2012; Moricová *et al.*, 2013; Wang *et al.*, 2015; Wang *et al.*, 2017; Yakovlev *et al.*, 2019). 5hmC has been reported in rice genomes but, in contrast to mammalian systems, it was found to occur preferentially in silenced transposable elements and heterochromatin regions (Wang *et al.*, 2015).

Kalinka *et al.* (2023) chose rye (*Secale* spp.) to investigate the presence of 5hmC and other 5mC oxidation products because rye genomes are enriched with heterochromatin regions and transposable elements. The study provided evidence of the presence of 5hmC in rye genomes and showed that the amounts of 5mC and 5hmC were correlated. It also revealed that, in contrast to rice, 5hmC was associated more closely with euchromatin than with heterochromatin.

## Detection of 5hmC and 5mC oxidative derivatives requires sensitive techniques

Various approaches have been employed to detect the global levels of 5hmC and other 5mC oxidation derivatives in genomes. The ability to detect these bases in plant genomes has been reported to be affected by the choice of detection method, partly because the amount of 5hmC is generally low. HPLC-MS with stable isotope reference compounds is the current ‘gold-standard’ approach for global 5hmC quantification. Comparatively, antibody-based approaches, such as immune-dot-blot and immunohistochemistry (IHC), are rapid and inexpensive but only semi-quantitative (Hack *et al.*, 2016). Scintillation counting using radiolabelled [^3^H]glucose has also been successfully implemented to assess 5hmC presence (Terragni *et al.*, 2012). In addition, to assay for 5mC and 5hmC at specific loci, affinity enrichment is done by immunoprecipitation and the resulting samples are used for high-throughput analysis. This method is known as methylated/hydroxy-methylated DNA immunoprecipitation (MeDIP HmeDIP). Bisulfite sequencing and digestion with methylation-sensitive restriction enzymes have also been used, but these methods fail to distinguish between 5mC and 5hmC (Yu *et al.*, 2012).

In their study, Kalinka *et al.* (2023) initially used ELISA to assess global levels of 5hmC. This method showed *S. sylvestre* to have higher levels of 5hmC than the other three species studied, but revealed no significant differences between tissue types. The presence of 5hmC (and other modifications) was then analysed by 2D-UPLC-MS/MS. A further analysis involved enrichment of methylated DNA using MeDIP and quantification using MS (2D-UPLC-MS/MS). This analysis provided the correlation between the occurrence of methylated cytosine and 5hmC or other modifications. Additionally, immunofluorescence was conducted to reveal the location of the 5hmC. MS also showed *S. sylvestre* to have the highest level of 5hmC, and revealed that the rye species with lower GC content had a higher cytosine methylation rate and, conversely, those with higher GC content had a lower cytosine methylation rate.

The study showed a high correlation between 5mC and 5hmC levels in different tissues of all the rye species, suggesting co-existence of the two modified bases. Furthermore, by comparing the chromosomal distribution of 5mC (Kalinka and Achrem, 2020) and 5hmC, Kalinka *et al.* (2023) were able to show that the two modified bases occur in the same regions of the chromosome. Importantly, both the modified bases were found to be absent in a large heterochromatin region, suggesting that 5hmC presence might be linked to coding rather than non-coding sequences in rye, in contrast to rice. Consistent with this, the ancestral rye species, *S. sylvestre*, showed a higher 5hmC content than the more recently evolved, cultivated species, *S. cereale* (rye evolution has been accompanied by genome enrichment with repetitive, non-coding sequences).

Kalinka *et al.* (2023) also tested for the presence of the 5hmC oxidized bases, 5fC and 5caC, and the deaminated base 5hmU, in the rye genomes. 5fC, 5caC, and 5hmU were all detected, although 5caC was found only at an extremely low level. Notably, high quantities of 5fC and 5hmU were observed in comparison with 5hmC, and a positive correlation was observed between 5mC and 5hmC/5fC, as well as between 5hmC and 5hmU. Interestingly, higher amounts of 5hmC, 5fC, and 5hmU were noted in the hypermethylated (5mC-enriched) DNA fraction, while 5caC was not detected, suggesting that 5caC occurs in 5mC-depleted regions. Furthermore, to exclude DNA damage as the cause of 5mC oxidative derivatives in rye genomes, Kalinka *et al.* (2023) measured the levels of uracil and 8oxoG (8-oxo-2’-deoxyguanosine; an oxygen damage marker). Lower correlations were noted between the 5mC derivatives and uracil or 8oxoG, appearing to rule out oxygen damage-induced derivatization.

## The presence of 5mC derivatives in plants is still an unresolved mystery

**Fig. 1.**
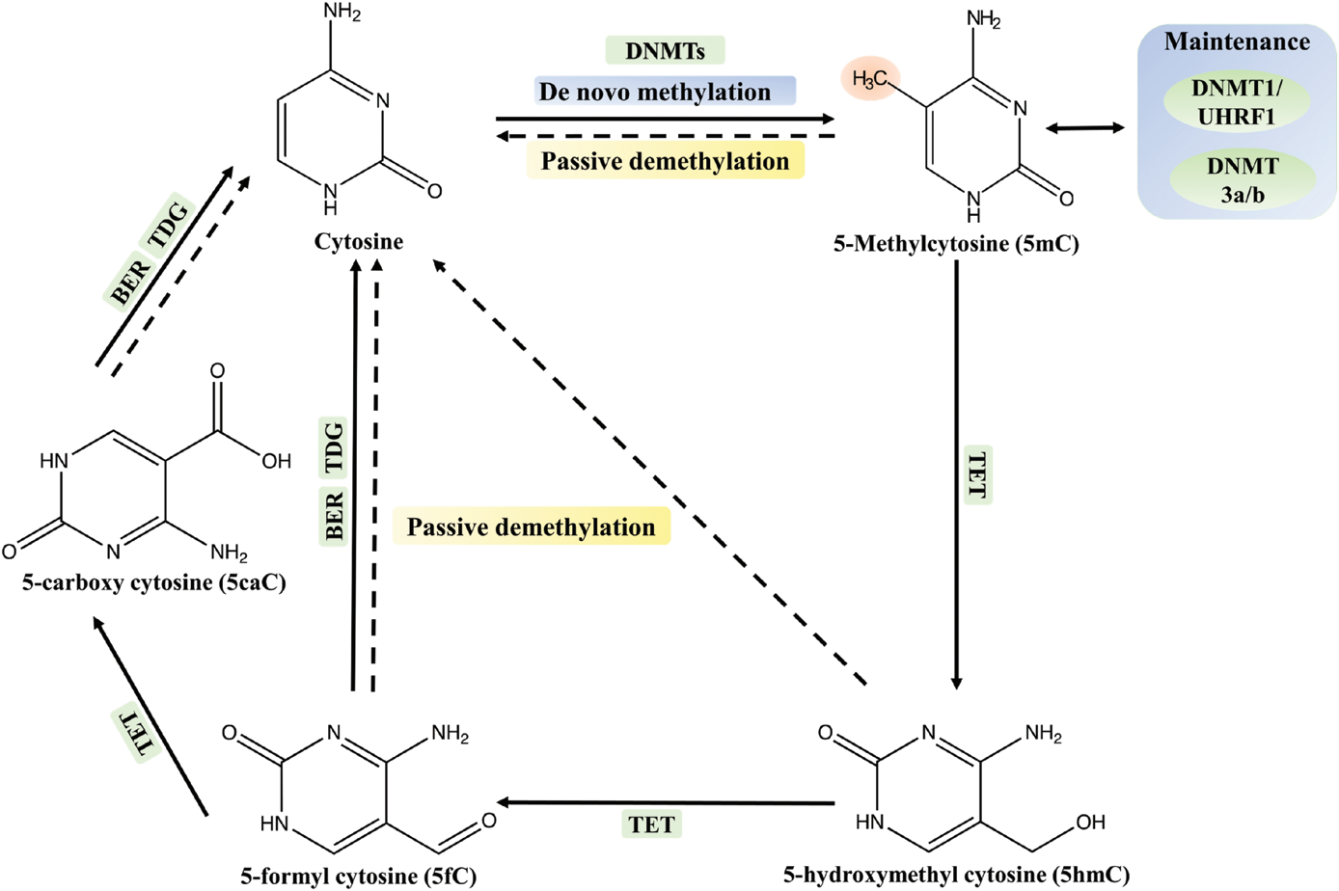
Overview of cytosine methylation/demethylation in mammals. Cytosine methylation occurs only at CG motifs and is mainly carried out by DNA methyltransferase enzymes known as DNMTs. The DNMT1/UHRF1 complex maintains methylation of DNA after DNA replication. Non-CG methylation occurs in pluripotent stem cells and neuron cells, and is maintained by DNMT3a and 3b demethylase enzymes. Demethylation of 5mC requires oxidation and/or deamination and is conducted by a family of dioxygenases known as ten–eleven translocation (TET) enzymes. TET enzymes hydroxylate 5mC to 5hmC, while oxidation of 5hmC via TET enzymes leads to 5-formylcytosine (5fC), and further oxidation of 5fC produces 5-carboxylcytosine (5caC). This deamination process creates a mismatch which is then recognized by the thymine-DNA glycosylase (TDG) and repaired by the base excision repair (BER) pathway. After Ji *et al.* (2016).

**Fig. 2.**
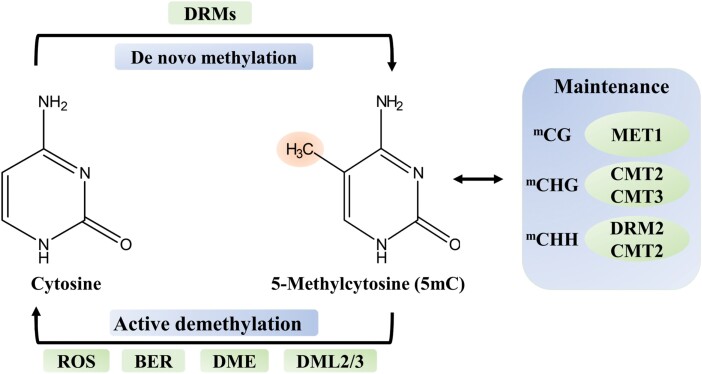
Overview of cytosine methylation/demethylation in plants. Cytosine methylation occurs at CG, CHG, and CHH motifs (where H represents A, T, or C). Cytosine methylation and maintenance involve different DNA methyl transferase enzymes, such as DNA methyl transferase (MET1), domain-rearranged methyl transferase (DRM), and chromo methyl transferase (CMT). MET1 methylates CG sites, while CMT3 and CMT2 methylate CHG and CHH motifs, respectively. DRM is involved in maintaining the existing methylation status as well as being responsible for *de novo* methylation. Demethylation of 5-methyl cytosine (5mC) can occur via active or passive mechanisms. The active mechanism is an enzymatic process which leads to 5mC removal, such as during DNA replication via base excision repair (BER). It mainly involves repressor of silencing 1 (ROS1), and demeter-like 2 or 3 (DML2/3) demethylases. These DNA demethylases can remove 5mC directly without the need for oxidation and/or deamination. Absence of functional DNA methylation maintenance, due to a lack of DNA methyltransferase activity, shortage of a methyl donor, or loss of methylation signal during successive rounds of replication, leads to passive loss of 5mC. Figure adapted from Zhang *et al.* (2018).

In vertebrates, TET family proteins are responsible for the hydroxylation of 5mC to form 5hmC and further oxidization of 5hmC to 5fC and 5caC (Fig. 1). However, there are no homologues of TET proteins in plants (Iyer *et al.*, 2009), while the DNA demethylases that are known to be present bring about direct removal of the methyl group on 5mC without oxidation or deamination (Fig. 2). On the other hand, the presence of 5mC derivatives cannot be the result of oxygen damage because the data on uracil and 8oxoG levels from Kalinka *et al.* (2023) are not consistent with that mechanism. In a previous report on Arabidopsis, mutation in a demethylase affected methylation only in a subset of specific loci (Lister *et al.*, 2008), suggesting that other demethylases and possibly other putative demethylation mechanisms must exist in plants and remain to be discovered.

## Future prospects

Although knowledge on epigenetic regulation in plants and the development of methods to detect epigenetic markers such as DNA methylation are increasing rapidly, there are still more questions yet to be answered. Most important are: what is performing the role of the mammalian TET proteins in plants, what is the mechanism behind 5mC derivatization, and how do 5mC derivatives regulate epigenetics? Also, how do 5mC oxidation-derived epigenetic markers change under biotic and abiotic stresses, and are they inherited from one generation to the next? Finding the answers to these questions would enable us to better understand plant genome regulation in response to different environmental conditions and inform the development and deployment of advanced genetic tools to enable agricultural sciences to combat impending climate change. Kalinka *et al.* (2023) provided convincing evidence to support the presence and probable regulatory role of 5mC derivatives in rye genomes, and this will open up pathways for further investigations into 5mC demethylation and its role in plant epigenetics.
